# Body mass index (BMI) influence on Cetuximab-induced antibody-dependent cellular cytotoxicity in advanced colon cancer

**DOI:** 10.1007/s11739-022-03124-4

**Published:** 2022-11-10

**Authors:** Antonino Carmelo Tralongo, Francesca Caspani, Ilaria Proserpio, Lisa Volpi, Leonardo Campiotti

**Affiliations:** 1 Medical Oncology Unit, ASST Settelaghi, University of Pavia, Varese, Italy; 2 Medical Oncology Unit, ASST Settelaghi, Varese, Italy; 3 Sub-Intensive Care Unit, ASST Settelaghi, Varese, Italy; 4 Department of Medicine and Surgery, University of Insubria, Viale Luigi Borri 57, 21100 Varese-Como, Italy

**Keywords:** Colorectal cancer, Metastatic, Cetuximab, BMI, Body weight

## Abstract

To date, we do not know if the excess of the body mass index (BMI) improves or worsens the outcomes in colorectal cancer treatment, and the correlation between BMI and prognosis remains unclear. A recent study in vitro showed a significant negative correlation between BMI and Cetuximab-induced antibody-dependent cellular cytotoxicity. On these bases, we tried to analyze the potential correlation between BMI and survival in patients affected by metastatic colorectal cancer (mCRC) and treated with Cetuximab. Retrospective data were collected from 132 patients affected by mCRC treated with Cetuximab in monotherapy or association with chemotherapy between January 2007 and October 2019. The cohort of patients was divided into different groups according to the World Health Organization (WHO) BMI classification: underweight (BMI < 18.59), normal weight (BMI 18.5–24.9,) overweight (BMI 25–29.9), and obese (BMI > 30), and we observed the influence of BMI on survival and treatment response. Patients with BMI ≥ 25 had statistically significantly better survival than patients BMI < 25 (19 vs 10 months, *p* = 0.025). Dividing the sample into the four WHO BMI categories, the best survival rates were seen in the overweight and obese subgroups (18 and 26 months respectively, *p* < 0.01). The multivariate analysis confirmed BMI as the only parameter able to influence survival. No correlation between BMI and treatment response was seen between BMI ≥ 25 and BMI ≤ 24 groups (*p* = 0.14). Our experience suggests that mild obese and overweight patients treated with Cetuximab could experience a better survival. We also observed that among normal weight, overweight, and mild obese patients, there is a better response to immunochemotherapy in comparison with underweight patients, but this difference does not reach a significative statistical value.

## Introduction

Colorectal cancer (CRC) is the most frequent tumor affecting gastrointestinal tract [1]. Several risk factors able to increase the likelihood of developing CRC have been found out in the last decades, related to genetic alterations, environmental exposure, and life-style [2]. Although inherited susceptibility is associated with the most prominent risk increasing, most CRC cases are sporadic due to unhealthy behavior during life. In particular, obesity emerged as a risk factor for CRC in several studies. Its rule seems to be involved even in the death rate, and bariatric surgery is associated with reducing the risk [3–5]. The prevalent localization of fat is critical. Visceral obesity is associated with various conditions that seem to be at the basis of the mechanisms of carcinogenesis [6]. It is explainable through at least three conditions related to the hallmarks of cancer:

1. The adipose tissue is associated with a high concentration of pro-inflammatory T helper cells (CD4 + and CD8 +), B cells, and dendritic and Natural Killer cells: the ideal microenvironment for the cancer development [7, 8].
2. The excess of insulin, promoting the production of insulin-like growth factor (IGF-1), contributes to the proliferation of cancer cells.
3. The increased output of adipokines7 enhances the pro-inflammatory microenvironment.

Therefore, the impact of obesity as a risk factor in the development of CRC is clear. However, several experiences suggest that exists a relationship between overweight and survival in CRC patients. Some studies demonstrated that overweight and early obese states are associated with improved survival, a phenomenon known as “obesity paradox”, more described in the cardiovascular and metabolic literature [9]. Others attributed to the high weight a worse prognosis.

To date, if the excess of body mass index (BMI) improves or gets worse, the outcomes are still controversial, and the correlation between BMI and prognosis remains unclear [10–12]. However, a recent study in vitro showed a negative effect of excess fatty tissue on Natural Killer (NK) cell’s ability to activate antibody-dependent cell cytotoxicity (ADCC) to colon cancer cell lines after exposure to Cetuximab [13]. Cetuximab is an EGFR-targeted monoclonal antibody. It showed clinical benefits as a component of treatment for patients with RAS wild-type metastatic colorectal cancer (mCR), becoming a mainstay in the clinical practice [14]. Indeed, in the setting of mCR, in addition to conventional chemotherapy drugs, several agents targeting the molecular drivers of CRC pathogenesis, including signaling pathways mediated by the epidermal growth factor receptor (EGFR) and vascular endothelial growth factor (VEGF), have been tested in such patients, increasing survival rates. In this background, we aimed to analyze the potential correlation between BMI and survival in those patients affected by mCRC and treated with Cetuximab.

## Methods

This observational retrospective multicentric “real life” study includes data extracted from medical records of patients suffering from mCRC treated with Cetuximab in monotherapy or association with chemotherapy from January 2007 to October 2019 in ASST Sette Laghi hospital, in Varese and Cittiglio centers, Italy. This study was approved by an internal ethics committee and was performed in accordance with the Declaration of Helsinki.

### Outcomes

The main aim of this study was to identify a potential correlation between BMI and survival in patients affected by mCRC. We also explored the influence of BMI on response treatment. Furthermore, we wanted to provide the distribution of the different BMI categories in a real-life population affected by mCRC and treated with Cetuximab.

### Patients

Data were retrospectively collected on mCRC patients according to the following inclusion criteria:

1. patients aged > 18 years
2. histopathologically confirmed diagnosis of colon carcinoma
3. K-RAS wild-type status of the tumor histopathological confirmed
4. pathological or radiological evidence of metastatic disease
5. patients treated or in treatment with Cetuximab
6. patients followed during their treatment in Varese and Cittiglio hospitals.

We did not analyze the patients with the following exclusion criteria:

1. presence of relevant comorbidity that makes the patients not eligible for the treatment
2. patients with another malignancy beyond mCRC
3. patients who not received at least four cycles of treatment
4. patients with infusion reaction during the first administration of Cetuximab.

### Data collection

As relevant characteristics of the sample, we collected: age, gender (male/female), and body mass index (BMI). Patients were divided into BMI ($\frac{{{\text{weight}}\left( {{\text{kg}}} \right)}}{{{\text{height}}\left( {\text{m}} \right)^{2} }}$) categories based on the WHO classification: underweight < 18.5, normal weight 18.5–24.9, overweight 25–29.9, and obese > 30. According to the principal international guidelines, the patient’s follow-up was performed by period clinical exams and radiological exams (mainly ultrasound, CT scan, and PET-FDG). The response of treatment was defined as complete response (CR), partial response (PR), or stable disease (SD) according to the Response Evaluation Criteria in Solid Tumors (RECIST criteria) 1.1.

### Statistical analysis

Data were summarized using descriptive statistics; median and range were reported for quantitative variables and absolute frequencies and percentages for categorical items. Differences between groups were evaluated with the Chi-square test, corrected with Yates correction. Survival curves were estimated with the Kaplan–Meier method. A univariate analysis was performed to explore association between a single patient characteristics and overall survival (OS). A multivariate Cox regression model was used to explore the association between OS and patient characteristics (BMI, age, and sex). Due to the small sample size, we have included few variables in this analysis. The first-degree error alpha was fixed to 0.05 bilaterally. Statistical analysis was performed using Statistical Package for Social Science SPSS version 25.

### Outcomes

The main aim of this study was to identify a potential correlation between BMI and survival in patients affected by mCRC. We also explored the influence of BMI on response treatment. Furthermore, we wanted to provide the distribution of the different BMI categories in a real-life population affected by mCRC and treated with Cetuximab.

## Results

### Patient characteristics

Applying the inclusion and exclusion criteria to the sample, we analyzed 132 patients. The median age was 67 (range 34–83). Men were more than women: 86 (65.2%) and 46 (34.8%), respectively. As expected in a real-life analysis, a good number of elderly patients (defined as > 65 years old) composed the sample (54%).

The patients were divided into four subgroups, based on the BMI categories: underweight (BMI < 20), normal weight (BMI 20–24.9), overweight (BMI  ≥ 25–29.9), and obese (BMI  ≥ 30). The predominant BMI subgroup was normal weight (43.9%), followed by the overweight subgroup (37.1%), obesity subgroup (12.9%), and underweight subgroup (6.1%) (Table 1). In the obesity subgroup, no patients belong to class II (BMI = 35–39) and class III (BMI  ≥ 40) obesity.

**Table 1:** Patients’ characteristics

Patients’ characteristics	
Age	
Median (range)	67 (34–83)
Patients ≥ 65 years, *n* (%)	72 (54.5)
Patients < 65 years *n* (%)	60 (45.5)
Gender	
Male *n* (%)	86 (65.2)
Female *n* (%)	46 (34.8)
BMI	
Median (range)	24.95 (16.7–40.8)
Underweight *n* (%)	8 (6.1)
Normal weight *n* (%)	58 (43.9)
Overweight *n* (%)	49 (37.1)
Obesity *n* (%)	17 (12.9)
Comorbidities and laboratoristic parameters’ serum albumin level before treatment (< 3.4 gr/L) *n* (%)	41 (31%)
Diabetes	21 (16%)
Hypertension	57 (43%)

*n* number

### Treatment characteristics and survival analysis

All patients were treated with Cetuximab in monotherapy or association with chemotherapy regimens approved by Drug Italian Agency (AIFA) for the treatment of mCRC. The best response to the treatment, defined as partial response (PR) or stable disease (SD), according to the RECIST criteria, was obtained in 60 (45.5%) patients; 60 (45.5%) patients had not any response to the Cetuximab’s therapy and showed a progression of disease. Data were not available for 12 (9.1%) patients for this analysis. The response’s duration was heterogeneous, in particular in a range of 2–21 months and a median of 8 months. Based on the BMI categories, only 2 (25% of the subgroup) underweight patients had a positive response to treatment (PR or SD) at first disease re-evaluation; 24 (42% of the subgroup) normal-weight patients had PR or SD; 26 (53.1% of the subgroup) overweight patients had PR or SD; 8 (47.1% of the subgroup) patients belong to obese subgroup had PR or SD. No data were available for this analysis in 6 (10.3%) patients, 5 (10%) patients, and 1 (5.9%) patients in the normal weight, overweight, and obese subgroups, respectively (Table 2).

**Table 2:** Treatment characteristics

Response to treatment in the entire cohort, *n* (%)	
PR or SD, *n* (%)	60 (45.5)
PD, *n* (%)	60 (45.5)
NA, *n* (%)	12 (9.1)
Response duration, *m* (range)	8 (2–21)^a^
Response to treatment based on BMI categories	
Underweight, *n* (%)	
RP or SD	2 (25)
PD	6 (75)
Normal weight	
RP or SD	24 (41.4)
PD	28 (48.3)
NA	6 (10.3)
Overweight	
RP or SD	26 (53.1)
PD	18 (36.7)
NA	5 (10.2)
Obese	
RP or SD	8 (47.1)
PD NA	8 (47.1) 1 (5.9)

^a^ = months

*n* number, *m* median, *NA* not available

**Fig. 1:**
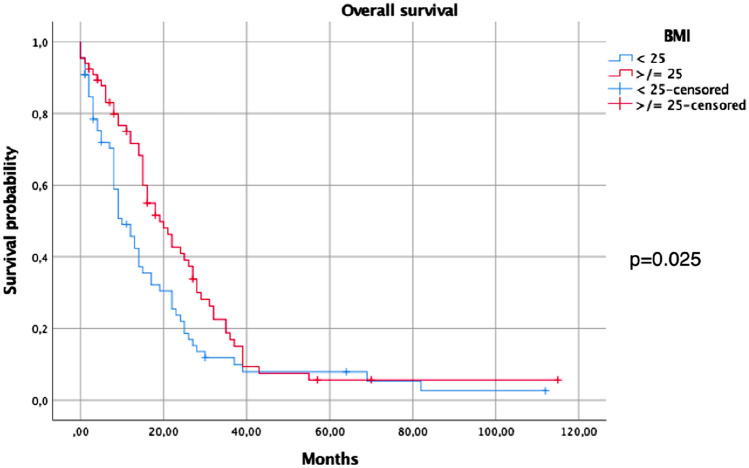
Overall survival among patients with BMI < or ≥ 25

**Fig. 2:**
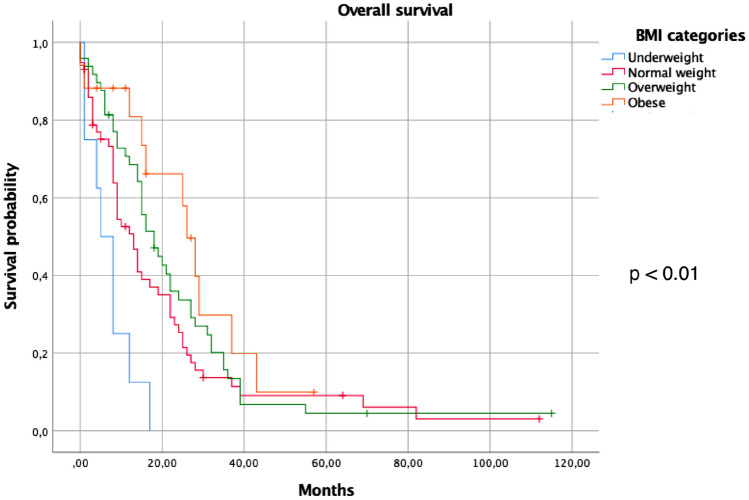
Overall survival according to different BMI subgroups

Dividing the sample into two groups, patients with BMI  ≥ 25 and patients with BMI ≤ 24, the analysis did not show a correlation between these groups and treatment response (*p* = 0.14). Univariate analysis for age and gender influence on survival was not significative (Table 3). Median Overall Survival (mOS) of the entire sample was 15 months (CI 95% 12.37–17.6). Considering the BMI subgroups, mOS was: 5 months (CI 95% 1.3–8.6) in the underweight group, 13 months (CI 95% 9.5–16.49) in the normal-weight group, 18 months (CI 95% 12.71–23.28) in the overweight group, and 26 months for the obesity one (CI 95% 21.54–30.45). The mOS was influenced by BMI: in particular, patients with BMI ≥ 25 had a statistically significant better survival than patients BMI < 25 (19 months CI 95% 13.91–24.08 vs 10 months, CI 95% 6.25–13.74, *p* = 0.025), (Fig. 1). These results were confirmed when the mOS was analyzed, dividing the sample into the different BMI categories, showing the best survival rates in the overweight and obese subgroups (18 and 26 months, CI 95% 1271–2328 and 21.54–30.45, respectively, *p* < 0.01), (Fig. 2). Exploring the potential influence of different factors on survival, the multivariate analysis confirmed BMI as the only parameter able to influence it (Table 4).

**Table 3:** Univariate analysis for all patients, exploring influence of different factors on survival

Variables	*P* value
Gender (male vs female)	0.31
Age ( ≥ 65 vs < 65)	0.62
BMI ( ≥ 25 vs < 25)	0.12

**Table 4:** Multivariate Cox regression analysis for all patients, exploring influence of different factors on survival

Variables	HR (CI 95%)	*P* value
Gender (male vs female)	0.71 (0.46–1.07)	0.10
Age ( ≥ 65 vs < 65)	1.08 (0.74–1.58)	0.67
BMI ( ≥ 25 vs < 25)	1.66 (1.12–2.46)	0.01

*P* < 0.05

*CI* confidence interval, *HR* hazard ratio

## Discussion

The relation between body weight and cancer survival is an important issue, increasingly studied in the last years. Awareness of this relationship, indeed, is essential during the diagnosis, when the clinician needs to communicate the prognosis, and during the treatment as well. Our experience suggests that the obese and overweight patients have better survival, confirming the results of the obesity paradox phenomenon: overweight and mild obesity, linked to a higher incidence of the epithelial tumor, are associated with better survival in patients affected by certain active cancer [9]. However, it is important to specify that the patients collected in the obesity subgroup belong to class I obesity. It means that better results in survival were shown just in those patients classified as mild obese. Therefore, we cannot assume that these advantages are kept even in severe obesity.

Moreover, it is interesting to see how the impact of BMI influences not only the survival but also the treatment response. From our results emerged a progressive better response when there is a progressive increase in body weight, although this result did not achieve a statistical significance (Table 2). These results agree with the experience of Shahjehan et al., where was identified as a positive correlation between BMI and 5-year survival in patients diagnosed with CRC. However, in this specific study, the results were achieved regardless of the treatment used [15].Nevertheless, this phenomenon is not found just in those patients affected by CRC. Several studies showed the same correlation in different histologies: renal cell carcinomas (surgically treated), acute myeloid leukemia in elderly patients, and lymphomas treated by autologous bone marrow transplant [16–19]. The explanation of these findings is still to clarify and remains controversial. The different fat distribution might interfere with the drug pharmacokinetic (chemotherapy agents, monoclonal antibody, or kinase inhibitor), influencing treatment outcomes [20]. Another possibility is due to an intrinsic characteristic of some histological subtypes, determining a better prognosis in the presence of high fat, as it is known in endometrial cancer and renal cell carcinoma [17]. The study by Campiotti et al. showed a significant negative correlation between body mass index and Cetuximab-induced antibody-dependent cellular cytotoxicity [13]. This is an in vitro study using blood from healthy donors; it may be that the known multiple and complex mechanisms underlying tumor growth and response to treatment have not intervened, thus giving different results.

It is equally valid that these results may belong to several biases. First of all, BMI is not the ideal measure of body adiposity, often confounding the lean mass and fat mass. Thus, a high BMI does not necessarily mean high fat, and to assert that obese patients, based on BMI classification, have a better survival, because the increased adiposity volume might be confusing. Another interesting point is the timing of BMI detection: a cachectic patient at diagnosis with high tumor burden certainly has a different prognosis than other patients with normal nutrition status and low tumor burden. In this case, better survival is not due to the different BMI, but of course, to other factors implied in the tumor aggressiveness. It is particularly relevant, because an underweight or normal weight might be an expression of some other occult factors able to modify the survival even more than the BMI. Although our exploratory analysis is following the literature on the same topic, it has some limitations. The sample contains a limited number of patients, and the retrospective nature of the study could inevitably influence it by a selection bias. Moreover, the results of the analysis could be influenced by the imbalance of the number of patients in the different BMI groups.

Besides, we could not retrieve data regarding diet, physical activity habits, and all those parameters able to influence the results of the correlation analyzed. Taking into consideration even others factors (lean and fat masses, muscle index, muscle density, subcutaneous fat index, subcutaneous fat density, visceral fat index, visceral fat density, etc.) may provide more and better answers.

## Conclusions

Mild obese and overweight patients treated with Cetuximab could experience a better survival. Moreover, we also observed that among normal weight, overweight, and mild obese patients, there is a better response to immunochemotherapy in comparison with underweight patients, but this difference does not reach a significative statistical value. Prospective studies would be of value to clarify relation between BMI and response to immunochemotherapy and the clinical role of BMI for survival in patients with mCRC.

## Abbreviations

BMI: Body mass index
mCRC: Metastatic colorectal cancer
CRC: Colorectal cancer
IGF-1: Insulin-like growth factor 1
NK: Natural killer
ADCC: Antibody-dependent cells cytotoxicity
EGFR: Epidermal growth factor receptor
VEGF: Vascular endothelial growth factor
CR: Complete response
PR: Partial response
SD: Stable disease
PD: Progressive disease
OS: Overall survival
mOS: Median overall survival
AIFA: Drug Italian agency
